# Evaluation of cardiovascular disease in patients with systemic arterial hypertension in relation to age and sex: a retrospective study in a south Indian population

**DOI:** 10.1590/1677-5449.010516

**Published:** 2017

**Authors:** Ashwini Aithal Padur, Aisyah binti Hamdan, Talissa Tatiana binti Intisar Patrick Abdullah, Chandrigga Gunalan, Naveen Kumar

**Affiliations:** 1 Manipal University, Melaka Manipal Medical College, Department of Anatomy, Manipal Campus, Manipal, Karnataka, India.; 2 Manipal University, Melaka Manipal Medical College, Manipal Campus, Manipal, Karnataka, India.

**Keywords:** hypertension, cardiovascular disease, risk factors, retrospective study, hipertensão, doença cardiovascular, fatores de risco, estudo retrospectivo

## Abstract

**Background:**

Systemic arterial hypertension manifests as constant elevation of blood pressure and is considered to be an important cardiovascular risk factor. Systemic evaluation of cardiovascular diseases in patients with systemic arterial hypertension is imperative for prevention.

**Objectives:**

The objective of the present study was to investigate and inter-relate sex and age with systemic arterial hypertension and cardiovascular diseases.

**Methods:**

Medical records of patients with systemic arterial hypertension and cardiovascular disease were evaluated. Data from the medical records were recorded in a prescribed protocol and the data were analyzed and correlated to the patients’ age and sex.

**Results:**

A total of 170 medical records for patients who visited the cardiology department were reviewed. Of these, 50 patients had systemic arterial hypertension and 19 of this subset had cardiovascular diseases. When we correlated systemic arterial hypertension with age, we observed that patients in the 51-60 years age group were more prone to systemic arterial hypertension (36%). Most of the patients with both systemic arterial hypertension and cardiovascular disease were female and in the 61-70 years age group, while among males the highest rate of occurrence was seen in the 51-60 years age group. The correlation coefficient (r) was 0.62, indicating a moderate, positive, linear relationship between systemic arterial hypertension and cardiovascular disease.

**Conclusions:**

A majority of patients with systemic arterial hypertension may develop cardiovascular disease and, as age increases, the tendency to develop hypertension also increases.

## Resumo

**Contexto:**

A hipertensão arterial sistêmica se manifesta como elevação constante da pressão sanguínea e é considerada um importante fator de risco cardiovascular. A avaliação sistêmica de doenças cardiovasculares em pacientes com hipertensão arterial sistêmica é crucial para a prevenção.

**Objetivos:**

O objetivo deste estudo foi investigar e inter-relacionar sexo e idade com hipertensão arterial sistêmica e doença cardiovascular.

**Métodos:**

Prontuários médicos de pacientes com hipertensão arterial sistêmica e doença cardiovascular foram avaliados. Dados dos prontuários médicos foram lançados em um protocolo pré-definido, e os dados foram analisados e correlacionados com a idade e o sexo dos pacientes.

**Resultados:**

Um total de 170 prontuários de pacientes que visitaram o departamento de cardiologia foram revisados. Destes, 50 pacientes apresentavam hipertensão arterial sistêmica, e destes, 19 tinham doença cardiovascular. Ao correlacionar hipertensão arterial sistêmica com idade, observamos que pacientes do grupo etário de 51-60 anos estavam mais suscetíveis a apresentar hipertensão arterial sistêmica (36%). A maioria dos pacientes com hipertensão arterial sistêmica e doença cardiovascular era do sexo feminino e estava no grupo etário de 61-70 anos; já entre os pacientes do sexo masculino, a maior taxa de ocorrência foi no grupo etário de 51-60 anos. O coeficiente de correlação (r) foi 0,62, indicando uma relação moderada, positiva, linear entre hipertensão arterial sistêmica e doença cardiovascular.

**Conclusões:**

A maioria dos pacientes com hipertensão arterial sistêmica pode desenvolver doença cardiovascular; conforme a idade aumenta, também cresce a tendência a desenvolver hipertensão.

## INTRODUCTION

Blood pressure is defined as the lateral pressure exerted by the column of blood on the walls of the arteries. It usually means arterial pressure, which is recorded in millimeters of mercury – for example 120/80 mmHg. The higher number denotes the systolic pressure while the lower number denotes the diastolic pressure.^1^ High blood pressure, or systemic arterial hypertension (SAH), is seen when blood pressure is constantly elevated and is considered an important cardiovascular risk factor. A study involving 52 countries all over the world showed that SAH is considered a greater relative risk factor for acute myocardial infarction than *diabetes mellitus* (DM).^2^ It has been observed that as age advances, the risk of developing SAH and cardiovascular disease (CVD) also increases. The blood vessels lose their flexibility with age, which can contribute to increased pressure throughout the system. Extensive epidemiologic studies have provided evidence that SAH accelerates development and progression of atherosclerosis, leading to CVD.

Both prevalence and incidence of CVD are increasing in the developing world.^3^ This may be due to the swift socioeconomic growth in developing countries and increasing exposure to CVD risk factors such as DM, SAH, hypercholesterolemia, and smoking. There are often no warning signs or symptoms of SAH and so many people are not aware of it. Although there have been improvements in prevention, treatment, and control of SAH, it still remains an important public health challenge. Onset of SAH and its effect on CVD may be modulated by various environmental and genetic factors. It has been postulated that socioeconomic, environmental, and genetic factors play an influential role in the development of CVD.^4^ The Indian population is believed to have higher risk and prevalence of CVD when compared to other ethnic groups.^5^ India is said to have 29.8 million symptomatic patients with CVD, 19.3 million diabetics, and 118 million hypertensive patients, who are at great risk of developing CVD.^6^ Despite the high rates of occurrence, there is very limited data regarding the correlation between the occurrence of CVD in hypertensive patients in India and its relation to sex and age.

The objectives of this study were therefore to investigate the occurrence of CVD in patients with SAH from a South Indian population, by retrospective evaluation, to investigate the relationships between sex, age, and SAH, and to inter-relate these factors with CVD.

## MATERIALS AND METHOD

### Data collection

This retrospective study was conducted at the Dr. T.M.A Pai Hospital, a primary tertiary care teaching hospital in Udupi district of Karnataka state, India. Human research ethics committee clearance was obtained prior to data collection (IEC 322/2015). Medical records from the past five years for patients with SAH who had CVD were collected from the medical records section of the hospital. Patients’ details were kept confidential and prior permission was obtained from the patients and their families. No interventions were performed. All relevant clinical and laboratory data was documented on a prescribed protocol. Patients’ demographic data, duration of hospital stay, type of CVD, whether or not they had diabetes, and smoking and drinking habits were noted. All relevant information was noted from eligible patients’ files based on the inclusion and exclusion criteria (Table 1). A total of 170 medical records for patients who had visited the cardiology department were reviewed.

**Table 1:** Inclusion and exclusion criteria for data collection.

**Inclusion criteria**	**Exclusion criteria**
Age under 70 years	Age over 70 years
Patients with hypertension	Patients without hypertension having cardiovascular disease
Patients having only cardiovascular diseases	Patients with any infectious disease and with congenital anomalies of the heart
Patients who are non-diabetic	Patients having cardiovascular diseases associated with other diseases

### Data analysis

The data collected were classified according to whether or not patients had SAH. Records for patients who had SAH and CVD were then selected and analyzed. They were then correlated to patient age and sex. A Spearman correlation coefficient [r] was calculated using the values of the two variables (SAH and CVD) to determine whether they were associated. The data were analyzed using SPSS (version 16.0) software.

## RESULTS

Of the total of 170 cases, 50 patients had SAH (29%) without any other illnesses diagnosed that are not related to cardiology and all were more than 20 years old. We excluded other records because the patients had SAH associated with other diseases. Of the remaining 50 patients, 27 were female and 23 were male.

**Figure 1:**
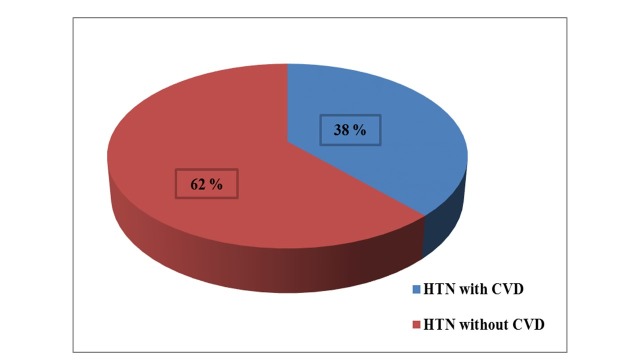
Relation between hypertension (HTN) and cardiovascular disease (CVD).

**Figure 2:**
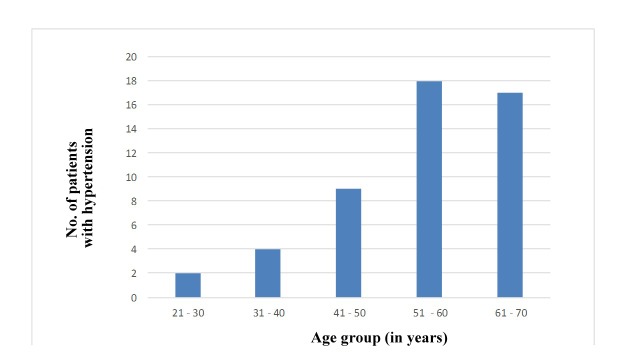
Graph showing relation (in percentage) between age and hypertension.

Nineteen of the patients with SAH had CVD (38%), while 31 of them did not have any CVD (62%) associated with SAH (Figure 1). This shows that SAH is one of the main risk factors of CVD. Upon evaluation of the trend of SAH against age, it was observed that patients in the 51-60 years age group were most prone to SAH (36%), followed by patients in the 61-70 years (34%), 41-50 years (18%), 31-40 years (8%), and 21-30 years (4%) age groups (Table 2 and Figure 2). This shows that the frequency of SAH increases as age advances.

**Table 2:** Relation between age and hypertension prevalence.

**Age (in years)**	**No. of patients with hypertension (n = 50)**
21-30	2 (4%)
31-40	4 (8%)
41-50	9 (18%)
51-60	18 (36%)
61-70	17 (34%)

**Figure 3:**
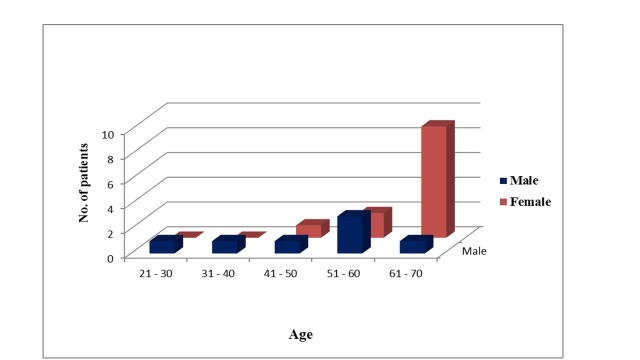
Graph showing the interrelation (in percentage) between hypertension (HTN) and cardiovascular disease (CVD), by patient age and sex.

When we related SAH to sex we observed that the prevalence was higher among females (54%) than among males (46%). Most of the female patients with SAH and CVD were in the 61-70 years age group, while among males the highest rate of occurrence was seen in the 51-60 years age group. The lowest frequency was seen in the 21-40 years age group (Figure 3 and Table 3).

**Table 3:** Interrelation between hypertension and cardiovascular disease, by patient age and sex.

**Sex**	**Age (in years)**					**Total number**
	**21-30**	**31-40**	**41-50**	**51-60**	**61-70**	
Male	1	1	1	3	1	7 (46%)
Female	0	0	1	2	9	12 (54%)

The Spearman correlation coefficient (r) for incidences of SAH and CVD showed a moderate, positive, linear relationship (r=2). This clearly indicates that SAH and CVD are significantly related to each other, which is a very important finding.

## DISCUSSION

Systemic arterial hypertension is diagnosed based on elevation of either systolic or diastolic blood pressure and the objective of hypertension management is to attain normalization of both. There is a strong and frequent association between SAH and CVD. Cardiovascular diseases are diseases in which several factors such as smoking, dyslipidemia, SAH, DM, obesity, and hereditary factors play major roles. According to reports, cardiovascular deaths cause 34% of global mortality in women and 28.2% of all deaths in men and are affect all sections of the society.^7^ It has been reported that CVD manifest almost a decade earlier in the Indian subcontinent when compared to the West.^8^ Additionally, it is stated that deaths related to CVD have been found to occur 5-10 years earlier in the Indian subcontinent than in Western countries.^9^ This shows that the rise in the prevalence of CVD is a reality that is becoming increasingly evident in India.

Systemic arterial hypertension is considered to be a major risk factor in recent years since patients with SAH have two times greater chances of getting CVD. In clinical practice, treatment of high blood pressure is focused on achieving health benefits for patients, whereas public health is focused on prevention of high blood pressure and to reduce the incidence of coronary heart disease in populations.^10^ A study by Razia et al., found that the percentage occurrence of CVD in the population studied was 57.3% and 42.2% of these were SAH patients.^11^ In our study, we found that 38% of the patients with SAH, had CVD, which demonstrates a high prevalence. The onset of SAH is mostly beyond the third decade of life; probably due to arterial stiffening and loss of arterial compliance that occurs with aging and other factors.^12^ This was also evident in our study, since we observed that patients in the 51-60 years age group are more prone to SAH (36%), followed by patients in the 61-70 years (34%) and 41-50 years (18%) age groups.

In terms of its relation to sex, while men are said to be predominantly affected by SAH, women are also no longer considered immune to its occurrence.^13^ According to Kannel, SAH was found to account for 39% of cases in men and 59% in women.^14^ Our observations were similar, since in our study population SAH associated with occurrence of CVD was higher in female patients (54%) than in male patients (46%) and the female patients were in the of 50-70 years age group. This is a very important observation and few studies have linked this occurrence to hormonal response. It has been stated that the risk of developing CVD in hypertensive compared with non-hypertensive individuals is about twofold in men and threefold in women.^15^ This is partly explained by early menopause in Indian women, which to a great extent reduces the protective effect of oestrogens.^16^

In recent years, infectious diseases have become relatively less of a concern, while chronic diseases like CVD continue to plague the global population. It has also been noted that most of these diseases are largely related to lifestyle factors, and can be minimized or prevented by lifestyle changes. This would be the most appropriate method to deal with SAH and CVD-related mortality and morbidity. Several evidence-based studies have constantly indicated a positive correlation between physical activity and good health. People who tend to have sedentary lifestyles risked an increase in blood pressure over time, whereas those who were physically active seemed to avoid this adverse effect.^17^ Implementing simple but effective strategies for prevention of CVD is conspicuously obvious. Therefore, there is very little actual research done in these areas in India.^18^^,^ 19 Preventive measures require multidisciplinary, multi-sectorial, and multi-level co-ordination and approaches that address the patient, provider, healthcare systems, public health, and public policy for the prevention and control of CVD in India.^20^

## CONCLUSION

The present study revealed that there exists a positive and significant relationship between CVD and SAH and that SAH is a major risk factor of CVD.
